# A Prospective Observational Study: Does Ramadan Fasting Worsen Rheumatoid Arthritis Disease Activity?

**DOI:** 10.7759/cureus.117341

**Published:** 2026-10-02

**Authors:** Chaimae Nouri, Latifa Tahiri, Meryem Kardoudi, Naima Chakyr, Fatine Kronbi, Hanan Rkain, Fadoua Allali

**Affiliations:** 1 Department of Rheumatology B, Ayachi Hospital, Mohammed V University, Faculty of Medicine and Pharmacy of Rabat, Rabat, MAR; 2 Physiology Laboratory, Mohammed V University, Faculty of Medicine and Pharmacy of Rabat, Rabat, MAR

**Keywords:** das28, disease activity, ramadan fasting, rheumatoid arthritis, treatment adherence

## Abstract

Introduction: The impact of Ramadan fasting on rheumatoid arthritis (RA) disease activity remains insufficiently documented, and available data are limited. This study aimed to assess the effect of Ramadan fasting on RA disease activity in patients deemed fit to fast. Secondary objectives were to explore factors potentially associated with worsening disease activity during Ramadan and to assess treatment adherence before and after Ramadan.

Methods: We conducted a prospective, observational, single-center study in the Rheumatology B Department of El Ayachi Hospital, Ibn Sina University Hospital Center, Rabat, Morocco, during Ramadan 2026. Patients over 18 years of age with RA meeting the American College of Rheumatology/European Alliance of Associations for Rheumatology (ACR/EULAR) classification criteria and deemed fit to fast were recruited consecutively during the month preceding Ramadan. Disease activity was assessed before and after Ramadan using clinical and biological parameters, including tender and swollen joint counts, morning stiffness, nocturnal awakenings, pain visual analog scale (VAS), fatigue, erythrocyte sedimentation rate (ESR), C-reactive protein (CRP), and DAS28-ESR. The primary endpoint was the change in 28-joint Disease Activity Score (DAS28) between the pre- and post-Ramadan assessments. An exploratory univariate analysis was performed to identify factors associated with worsening disease activity. Treatment adherence was assessed before and after Ramadan using the four-item Morisky-Green-Levine scale.

Results: A total of 50 patients were included in the study. The mean age was 55.96 ± 10.40 years, and 44 patients (88.0%) were women. No statistically significant change in overall disease activity was observed before and after Ramadan. DAS28 changed from 2.85 ± 1.28 to 2.96 ± 1.31 (p = 0.481). No significant differences were found in pain VAS, nocturnal awakenings, fatigue, morning stiffness, tender joint count, swollen joint count, ESR, or CRP. Overall, 38 patients (76.0%) showed no worsening of disease activity, while 12 patients (24.0%) showed worsening. Worsening was associated with longer RA duration (18.13 ± 6.49 vs. 12.54 ± 9.77 years; p = 0.023) and lower baseline DAS28 (2.33 ± 0.81 vs. 3.01 ± 1.16; p = 0.031). High treatment adherence was observed in 47 patients (94.0%) before Ramadan and 46 patients (92.0%) after Ramadan.

Conclusion: Ramadan fasting was not associated with a statistically significant worsening of RA disease activity. Most patients remained clinically stable, and treatment adherence remained high before and after Ramadan. These findings suggest that Ramadan fasting may be well tolerated in patients with RA who are deemed fit to fast and receive appropriate clinical follow-up.

## Introduction

Ramadan corresponds to the ninth month of the Islamic lunar calendar. During this period, Muslims abstain from dawn to sunset from all food and fluid intake, as well as from certain physical needs, such as smoking and sexual intercourse. This fast also carries an important spiritual dimension. Food intake is permitted before dawn and after sunset. The month lasts 29-30 days depending on the observation of the lunar crescent, and the daily duration of the fast varies according to season and latitude, averaging approximately 13 hours and reaching up to 18 hours in some regions. Although prescribed for the majority of adult Muslims, exemptions are granted to certain categories of individuals, including pregnant, breastfeeding, or menstruating women, travelers, individuals with conditions likely to be aggravated by fasting, and prepubertal subjects and frail elderly individuals [1].

Ramadan fasting induces several physiological adaptations, including metabolic, circadian, and immuno-inflammatory changes. Available data generally suggest a decrease in certain inflammatory markers, an improvement in some metabolic and vascular parameters, as well as changes in the gut microbiota, with no demonstrated deleterious metabolic effect in healthy subjects. These changes could thus influence the activity of certain inflammatory diseases [1,2].

Rheumatoid arthritis (RA) is a chronic systemic autoimmune disease characterized by persistent inflammation of the synovial joints, which can progress to joint destruction and impaired quality of life. As such, RA represents both a major clinical concern and a significant public health issue [3,4].

Although several studies have examined the effect of intermittent fasting on inflammatory markers, data regarding its specific impact on RA disease activity remain limited and heterogeneous. Some studies suggest a favorable effect, while others show no clearly established specific benefit [5-8]. Moreover, patients frequently express concerns about a possible worsening of joint symptoms during this period. In this context, the primary objective of our study was to assess the impact of Ramadan fasting, considered a particular form of intermittent fasting, on RA disease activity. The secondary objectives were to explore factors potentially associated with worsening disease activity during Ramadan and to assess patients' treatment adherence before and after Ramadan.

## Materials and methods

This was a prospective, observational, single-center study conducted in the Rheumatology B Department of El Ayachi Hospital, Ibn Sina University Hospital Center, Rabat, Morocco, from January 19 to March 29, 2026, covering the period preceding Ramadan, Ramadan 2026 itself, and the immediate post-Ramadan period.

We included patients older than 18 years with RA fulfilling the 2010 American College of Rheumatology/European League Against Rheumatism (ACR/EULAR) classification criteria [9], deemed fit to fast, and recruited consecutively among patients seen as outpatients or hospitalized in the Rheumatology B Department during the month preceding Ramadan 2026. We excluded patients who had received a corticosteroid bolus within the three months preceding the study, those who had undergone a recent change in RA treatment, and patients deemed unfit to fast.

Data were collected through face-to-face interviews using a questionnaire administered by a rheumatologist at two time points: during the month preceding Ramadan and within the week following its end. A Ramadan-specific questionnaire was developed by the authors based on a review of the literature and informed by previously published studies evaluating Ramadan fasting in patients with rheumatic diseases [1,6,8]. The questionnaire was pilot-tested in five patients before use. The complete author-developed Ramadan-specific questionnaire is provided in the Appendix. The collected data included sociodemographic characteristics, comorbidities, disease characteristics, current treatments, treatment adherence, treatment tolerability, and patients' knowledge and perceptions regarding the impact of Ramadan on RA activity.

Sociodemographic data were obtained from medical records. Comorbidities, RA-related characteristics, and current treatments were collected from medical records and supplemented by patient interviews. Treatment adherence was assessed using the four-item Morisky-Green-Levine Medication Adherence Questionnaire (MGL MAQ) [10]. Each "yes" response was assigned 1 point, and each "no" response was assigned 0 points, yielding a total score ranging from 0 to 4. Patients were classified as having high adherence with a score of 0, moderate adherence with a score of 1 or 2, and non-adherence with a score of 3 or 4. Patients' knowledge and perceptions regarding the impact of Ramadan on their disease were assessed through interviews.

Disease activity was assessed before and after Ramadan by a rheumatologist using clinical and biological parameters obtained through patient interviews, clinical examination, and review of medical records. Clinical parameters included the swollen joint count, tender joint count, duration of morning stiffness, nocturnal awakenings, pain intensity assessed using a visual analog scale (VAS) [11], fatigue, and patient global assessment of disease activity. Biological parameters included erythrocyte sedimentation rate (ESR) and C-reactive protein (CRP), obtained either from the medical record or measured systematically during the month preceding Ramadan and within the week following its end. The 28-joint Disease Activity Score based on erythrocyte sedimentation rate (DAS28-ESR) was calculated for each patient as previously described by Prevoo et al. [12]. The primary endpoint was the change in DAS28-ESR between the pre-Ramadan and post-Ramadan assessments. An increase in DAS28-ESR of >0.6 was considered worsening, a decrease of >0.6 was considered improvement, and changes between −0.6 and +0.6 were considered stable. For exploratory analysis, stable and improved patients were combined into a "no worsening" group.

Statistical analysis was performed using Statistical Product and Service Solutions (SPSS, version 23; IBM SPSS Statistics for Windows, Armonk, NY). Quantitative variables were expressed as mean ± standard deviation or as median with interquartile range, depending on their distribution, and qualitative variables as numbers and percentages. Comparisons of parameters before and after Ramadan were performed using the paired Student's t-test for normally distributed data or the Wilcoxon signed-rank test otherwise. For exploratory comparisons between the worsening and no-worsening groups, the independent-samples Student's t-test or Mann-Whitney U test was used for quantitative variables, as appropriate, and the chi-square test or Fisher's exact test for categorical variables. The significance threshold was set at p < 0.05.

Ethical and regulatory considerations were addressed before the start of the study in accordance with the ethical principles of the Declaration of Helsinki and its subsequent amendments [13]. The study was approved by the Comité d’Ethique pour la Recherche Biomédicale (CERB) (approval/reference number: 141/26). Participants were informed of the study objectives, data collection procedures, and the voluntary nature of participation. Informed consent was obtained from all participants before inclusion, and anonymity and confidentiality were maintained throughout the study.

## Results

A total of 50 patients were included in the study and completed the questionnaire. The mean age was 55.96 ± 10.40 years, and 44 (88.0%) participants were women. The mean duration of rheumatoid arthritis was 13.88 ± 9.35 years, and 34 (68.0%) had seropositive RA. Most patients lived in an urban setting (42, 84.0%) and received regular rheumatology follow-up (48, 96.0%). Arterial hypertension was the most frequent comorbidity, affecting 12 (24.0%) patients.

Regarding treatment, methotrexate was the most commonly used conventional synthetic disease-modifying antirheumatic drug (csDMARD), prescribed to 29 (58.0%) patients. Among biologic therapies, 15 (30.0%) patients received an anti-CD20 agent, 13 (26.0%) an anti-TNFα agent, and 7 (14.0%) an anti-IL-6 agent. Concomitant corticosteroid therapy was used by 37 (74.0%) patients. The demographic, clinical, socioeconomic, and treatment characteristics of the study population are presented in Table 1.

**Table 1:** General characteristics of the study population Quantitative variables are presented as mean ± standard deviation, and qualitative variables as number (%). csDMARDs: conventional synthetic disease-modifying antirheumatic drugs

Parameter	Result
Demographic data	
Female sex	44 (88.0)
Age (years)	55.96 ± 10.40
Disease characteristics	
RA disease duration (years)	13.88 ± 9.35
Seropositive RA	34 (68.0)
Regular rheumatology follow-up	48 (96.0)
Socioeconomic data	
Urban residence	42 (84.0)
Married	27 (54.0)
Unemployed/no occupation	36 (72.0)
Illiterate	22 (44.0)
Comorbidities	
Arterial hypertension	12 (24.0)
Treatments	
Corticosteroid therapy	37 (74.0)
csDMARDs	
Methotrexate	29 (58.0)
Leflunomide	11 (22.0)
Sulfasalazine	3 (6.0)
bDMARDs	
Anti-CD20	15 (30.0)
Anti-TNFα	13 (26.0)
Anti-IL-6	7 (14.0)

All patients had previously fasted during Ramadan after their rheumatoid arthritis diagnosis. The mean number of fasting days during the current study was 27.26 ± 5.44 days, accounting for mandatory interruptions, such as menstruation. Forty-five (90.0%) patients reported having previously received information about rheumatoid arthritis and Ramadan, with the treating physician being the main source of information (41, 82.0%), followed by the media (2, 4.0%) and word of mouth (2, 4.0%). During Ramadan, 42 (84.0%) patients adjusted the timing of their medication intake according to medical advice, while eight (16.0%) maintained their usual treatment schedule. No patient reported spontaneous dose reduction or treatment discontinuation during the fasting month. Increased use of symptomatic treatment was reported by only two (4.0%) patients. Treatment adherence remained high before and after Ramadan, with high adherence observed in 47 (94.0%) and 46 (92.0%) patients, respectively.

Regarding patients' perceptions, 30 (60.0%) believed that fasting improved their rheumatic condition, while 3 (6.0%) believed that fasting could trigger a disease flare. In addition, 45 (90.0%) patients considered Ramadan fasting compatible with both rheumatoid arthritis and antirheumatic treatment. Ramadan-related characteristics, treatment adaptation, treatment adherence, and patients' perceptions are summarized in Table 2.

**Table 2:** Ramadan-related characteristics, treatment adherence, and patient perceptions Quantitative variables are presented as mean ± standard deviation, and qualitative variables as number (%). Percentages for sources of information were calculated using the total study population (N = 50) as the denominator. RA: rheumatoid arthritis.

Parameter	Result
Fasting-related data	
Previous Ramadan fasting after RA diagnosis	50 (100.0)
Number of fasting days during the current study	27.26 ± 5.44
Patient information and knowledge	
Previously received information on RA and Ramadan	45 (90.0)
Source of information: treating physician	41 (82.0)
Source of information: media	2 (4.0)
Source of information: word of mouth	2 (4.0)
Treatment adaptation during Ramadan	
Usual treatment schedule maintained	8 (16.0)
Medication timing changed according to medical advice	42 (84.0)
Spontaneous dose reduction	0 (0.0)
Treatment discontinuation	0 (0.0)
Increase in symptomatic treatment during Ramadan	2 (4.0)
Treatment adherence (Morisky-Green-Levine Medication Adherence Questionnaire)	
High adherence before Ramadan	47 (94.0)
Moderate adherence before Ramadan	3 (6.0)
Non-adherence before Ramadan	0 (0.0)
High adherence after Ramadan	46 (92.0)
Moderate adherence after Ramadan	4 (8.0)
Non-adherence after Ramadan	0 (0.0)
Patient beliefs and perceptions	
Fasting may trigger a disease flare	3 (6.0)
Fasting improves the rheumatic condition	30 (60.0)
Fasting is compatible with RA	45 (90.0)
Fasting is compatible with antirheumatic treatments	45 (90.0)

Assessment of disease activity before and after Ramadan showed no statistically significant differences across the parameters studied. Some clinical parameters, including pain VAS, nocturnal awakenings, fatigue VAS, and morning stiffness duration, showed slight but non-significant improvement. Inflammatory markers, including ESR and CRP, also showed no statistically significant change. Regarding the primary endpoint, the DAS28-ESR changed from 2.85 ± 1.28 before Ramadan to 2.96 ± 1.31 after Ramadan, with no statistically significant difference (p = 0.481). The distribution of patients across DAS28-ESR disease activity categories also remained largely stable before and after Ramadan. Changes in clinical and biological disease activity parameters are presented in Table 3, and the distribution across DAS28-ESR categories is illustrated in Figure 1.

**Table 3:** Evolution of disease activity before and after Ramadan Quantitative variables are expressed as mean ± standard deviation or median (Q1-Q3), depending on their distribution. VAS: visual analog scale; ESR: erythrocyte sedimentation rate; CRP: C-reactive protein; DAS28-ESR: 28-joint Disease Activity Score based on erythrocyte sedimentation rate

Parameter	Before Ramadan	After Ramadan	p
Pain VAS	2.60 ± 2.22	2.08 ± 2.19	0.125
Nocturnal awakenings	0.00 (0.00-2.00)	0.00 (0.00-1.00)	0.339
Fatigue VAS	2.00 (1.00-4.00)	1.00 (0.00-4.00)	0.199
Morning stiffness (min)	0.00 (0.00-10.00)	0.00 (0.00-0.00)	0.147
Tender joint count	0.00 (0.00-5.25)	0.00 (0.00-4.25)	0.932
Swollen joint count	0.00 (0.00-2.00)	0.00 (0.00-2.00)	0.968
ESR (mm/h)	18.50 (7.75-35.25)	20.00 (8.75-37.50)	0.452
CRP (mg/L)	2.80 (1.35-11.00)	3.35 (1.28-8.48)	0.252
DAS28-ESR	2.85 ± 1.28	2.96 ± 1.31	0.481

**Figure 1:**
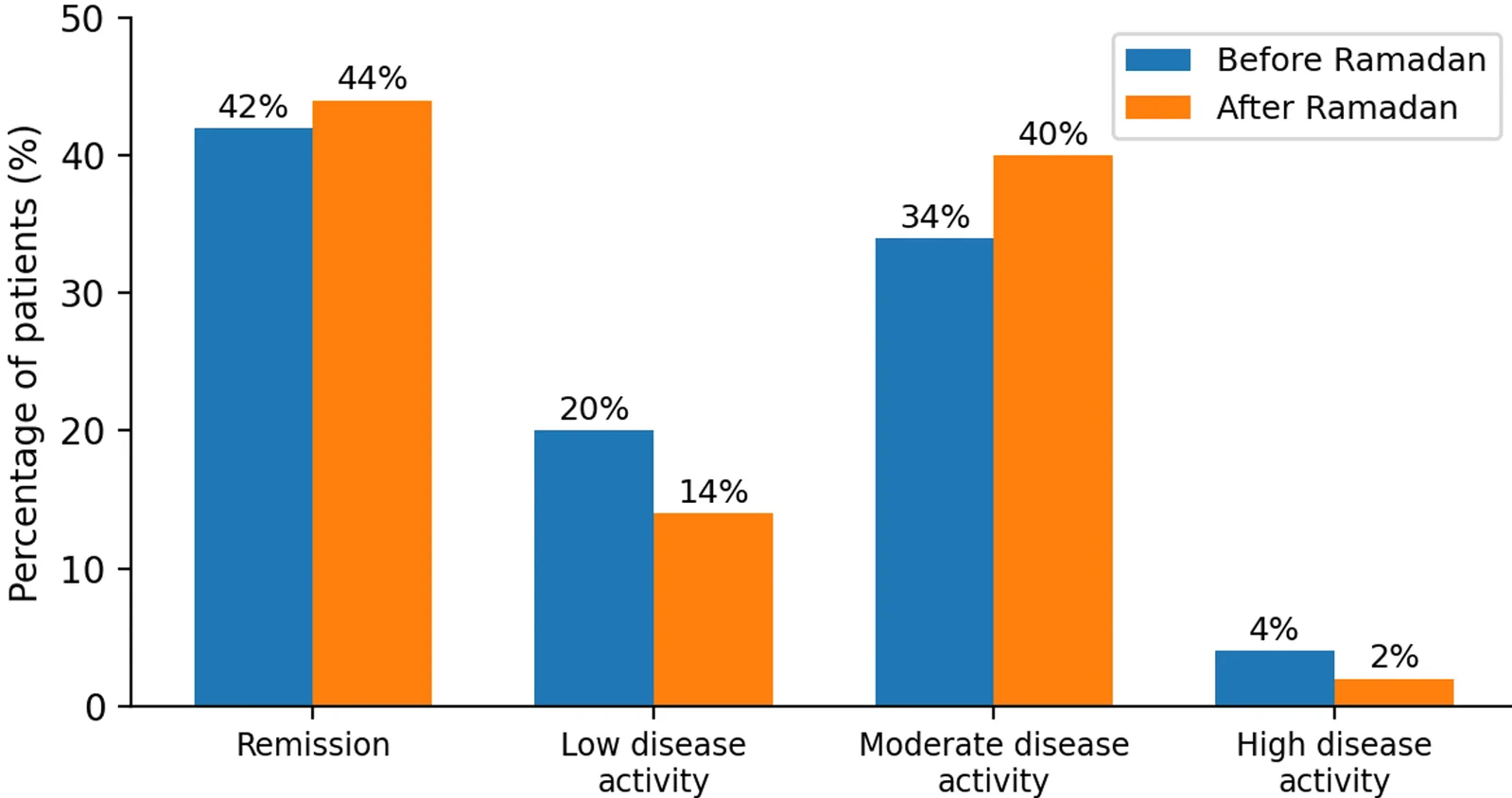
Distribution of patients according to DAS28-ESR disease activity category before and after Ramadan The distribution of patients across DAS28-ESR disease activity categories remained broadly stable between the pre-Ramadan and post-Ramadan assessments. DAS28-ESR: 28-joint Disease Activity Score based on erythrocyte sedimentation rate

To explore factors potentially associated with worsening disease activity during Ramadan, patients were divided into two groups according to the change in DAS28-ESR between the pre- and post-Ramadan assessments: a worsening group (n = 12; 24.0%) and a no-worsening group, comprising patients who remained stable or improved (n = 38; 76.0%). Univariate analysis showed that RA disease duration was significantly longer in the worsening group than in the no-worsening group (18.13 ± 6.49 vs. 12.54 ± 9.77 years; p = 0.023). Conversely, baseline DAS28-ESR was significantly lower in the worsening group (2.33 ± 0.81 vs. 3.01 ± 1.16; p = 0.031).

No statistically significant associations were found with age, sex, serological status, corticosteroid therapy, methotrexate use, biologic therapy, number of fasting days, change in medication timing, request for medical information, or perception of a beneficial effect of fasting. The results of the univariate analysis are presented in Table 4.

**Table 4:** Univariate analysis of factors associated with worsening rheumatoid arthritis disease activity during Ramadan Quantitative variables are expressed as mean ± standard deviation and qualitative variables as n (%). DAS28-ESR: 28-joint Disease Activity Score based on erythrocyte sedimentation rate; RA: rheumatoid arthritis

Variable	No worsening (n = 38)	Worsening (n = 12)	p
Age (years)	57.16 ± 9.78	52.17 ± 11.78	0.131
RA disease duration (years)	12.54 ± 9.77	18.13 ± 6.49	0.023
Baseline DAS28-ESR	3.01 ± 1.16	2.33 ± 0.81	0.031
Female sex	33 (86.8)	11 (91.7)	1.000
Seropositive RA	26 (68.4)	8 (66.7)	0.398
Corticosteroid therapy	29 (76.3)	8 (66.7)	0.707
Methotrexate	24 (63.2)	5 (41.7)	0.314
Biologic therapy	28 (73.7)	7 (58.3)	0.471
Number of fasting days	27.89 ± 4.34	25.25 ± 7.93	0.125
Change in medication timing	32 (84.2)	10 (83.3)	1.000
Information requested from physician	6 (15.8)	5 (41.7)	0.105
Fasting improves the disease	24 (63.2)	6 (50.0)	0.506

## Discussion

In our study, Ramadan fasting was not associated with a statistically significant change in RA disease activity. DAS28-ESR, joint indices, ESR, and CRP remained largely stable, and 76.0% of patients showed no worsening of disease activity. These findings are consistent with those of Siddique et al. [7] and Ben Nessib et al. [8], who reported stability or transient improvement in RA activity during Ramadan [6-8]. A recent review focusing on fasting and caloric restriction in RA similarly concluded that short-term effects are generally neutral or favorable while emphasizing the heterogeneity of study protocols, small sample sizes, and the limited overall level of evidence [5]. In our cohort, this apparent good tolerance may also be related to the selection of patients deemed fit to fast, regular rheumatology follow-up, high treatment adherence, and the absence of recent therapeutic changes.

Several immunometabolic mechanisms may contribute to this stability. RA is characterized by persistent activation of both the innate and adaptive immune systems, with increased production of pro-inflammatory cytokines, notably tumor necrosis factor-alpha (TNF-α), interleukin-1 beta (IL-1β), and interleukin-6 (IL-6), which contribute to synovial inflammation and joint destruction [3,4]. Intermittent fasting induces prolonged periods without caloric intake, promoting mobilization of lipid stores and metabolic changes that may modulate inflammatory pathways. A recent meta-analysis of 21 studies reported reductions in TNF-α, CRP, and leptin with intermittent fasting, with no significant changes in IL-6 or adiponectin [14]. These findings suggest a potential, although not uniform, anti-inflammatory effect that may vary according to the type and duration of fasting, associated weight changes, and the metabolic characteristics of participants.

Circadian rhythms play an important role in the pathophysiology of RA. Pain, stiffness, and joint swelling are often more pronounced upon waking and have been associated with nocturnal and early-morning increases in pro-inflammatory cytokines, particularly IL-6 [15-17], together with a relatively insufficient cortisol response [18]. During Ramadan, changes in meal timing, waking for suhoor, and sleep fragmentation may disrupt circadian rhythms and influence fatigue, pain, and morning stiffness without necessarily reflecting an inflammatory flare. In our study, the stability of joint indices and inflammatory markers suggests that some symptom variations may reflect circadian and behavioral adaptations rather than increased inflammatory activity.

Dietary intake between iftar and suhoor may modulate the potential effects of fasting. A diet rich in sugary, refined, and high-fat foods may promote an unfavorable metabolic and inflammatory profile, whereas Mediterranean-style or predominantly plant-based dietary patterns, rich in vegetables, legumes, whole grains, nuts, and olive oil, have been associated with improvement in certain RA symptoms and inflammatory parameters [19,20]. Ramadan fasting should therefore not be considered simply as caloric restriction, as its effects may also depend on the quantity and quality of food consumed during the nighttime period.

This issue is particularly relevant in the Moroccan context. A study conducted among 340 urban and rural households in the Rabat-Salé-Kénitra region reported that, during Ramadan, food expenditure increased by approximately 50%, together with significant increases in energy, carbohydrate, sucrose, and sodium intake and a decrease in protein intake [21]. These findings illustrate the coexistence of daytime fasting with nighttime meals that may be more abundant and richer in sugary or refined foods. The quality of food consumed between iftar and suhoor may therefore be as important as the duration of fasting itself. The absence of detailed dietary data in our study prevented us from exploring this potential interaction.

The gut microbiota may represent a link between meal timing, dietary composition, and inflammation. Ramadan fasting and time-restricted eating have been associated with changes in gut microbial diversity and composition, although findings in humans remain heterogeneous and their clinical significance is still uncertain [22]. These effects may depend partly on dietary quality, suggesting that they cannot be attributed to fasting alone. In RA, this mechanism represents an interesting area for future research.

Finally, our exploratory analysis found that worsening was more frequent among patients with longer disease duration and lower baseline DAS28-ESR. These findings should be interpreted with caution given the small sample size, the limited number of worsening events, spontaneous fluctuations in disease activity, and the limitations of DAS28-based measures in assessing individual changes [23]. Lower baseline disease activity may also make modest score variations appear more pronounced without necessarily corresponding to a clinically meaningful flare.

Strengths and limitations

This study has several strengths. Its prospective design allowed the same patients to be evaluated before and after Ramadan using both clinical and biological measures of RA disease activity, including DAS28-ESR, joint counts, ESR, and CRP. Treatment adherence and Ramadan-related behaviors and perceptions were also assessed, providing a broader evaluation of fasting in a real-world clinical setting.

Nevertheless, several limitations should be acknowledged. The single-center design and relatively small sample size may limit the generalizability of the findings and the statistical power of the exploratory analyses. In addition, the inclusion of patients deemed fit to fast may have introduced selection bias and limited the applicability of our findings to patients with more severe disease or contraindications to fasting. The absence of a non-fasting control group prevents us from distinguishing changes specifically attributable to Ramadan fasting from the natural variability of RA disease activity. The short follow-up period also precludes assessment of longer-term effects. Finally, detailed data on dietary intake, sleep patterns, body weight changes, and inflammatory cytokines were not collected, and disease activity assessment did not include imaging evaluation of synovitis by ultrasonography or magnetic resonance imaging, preventing evaluation of these potential influences and subclinical inflammatory changes during Ramadan.

## Conclusions

In our study, Ramadan fasting was not associated with a statistically significant worsening of RA disease activity. Most patients showed no worsening during this period, with good treatment adherence and appropriate adjustment of medication schedules. These findings suggest that, among patients with RA deemed fit to fast and appropriately monitored, Ramadan fasting appears to be generally well tolerated in the short term. Nevertheless, fasting decisions should remain individualized and take into account baseline disease activity, disease duration, current treatment, comorbidities, and the patient's overall clinical condition.

## Appendices

Study questionnaire

Pre-Ramadan Assessment

We will assess disease activity, treatment tolerance and adherence, and patients' knowledge regarding the effect of Ramadan on their rheumatoid arthritis.

General Information

Sex

☐ Female   |   ☐ Male

Age (years): ______________________________________________

Marital status - single response

☐ Single   |   ☐ Married   |   ☐ Divorced   |   ☐ Widowed

Educational level - single response

☐ Illiterate   |   ☐ Primary school   |   ☐ Middle school   |   ☐ High school   |   ☐ University

Place of residence - single response

☐ Urban   |   ☐ Rural

Health coverage - single response

☐ AMO Solidaire   |   ☐ CNOPS   |   ☐ CNSS   |   ☐ Private insurance   |   ☐ CNOPS student   |   ☐ No coverage

Current occupation - single response

☐ Student   |   ☐ Manual worker   |   ☐ Employee   |   ☐ Technician   |   ☐ Executive   |   ☐ Retired   |   ☐ Self-employed   |   ☐ Unemployed

Comorbidities - multiple responses possible

☐ Hypertension   |   ☐ Diabetes   |   ☐ Thyroid disorder   |   ☐ Kidney disease   |   ☐ Dyslipidemia   |   ☐ Malignancy   |   ☐ Crohn’s disease   |   ☐ Ulcerative colitis   |   ☐ Chronic H. pylori gastritis   |   ☐ Gastroesophageal reflux disease   |   ☐ Psoriasis   |   ☐ Never followed   |   ☐ Other: __________

Toxic habits - multiple responses possible

☐ Smoking   |   ☐ Alcohol use   |   ☐ None

Information on Rheumatoid Arthritis

Type of rheumatoid arthritis - single response

☐ Seropositive   |   ☐ Seronegative

History of Inflammatory Rheumatic Disease

Duration of osteoarticular symptoms related to the rheumatic disease (months): ______________________________________________

Duration of rheumatology follow-up (months): ______________________________________________

Do you have regular follow-up with your rheumatologist? - single response

☐ Yes   |   ☐ No   |   ☐ Yes, but I sometimes miss appointments

Current Treatment

csDMARDs - multiple responses possible

☐ Methotrexate   |   ☐ Sulfasalazine   |   ☐ Leflunomide

Methotrexate dose (mg/week) - single response

☐ 10   |   ☐ 12.5   |   ☐ 15   |   ☐ 17.5   |   ☐ 20   |   ☐ 22.5   |   ☐ 25

Route of administration - single response

☐ Oral   |   ☐ Injectable

Biologic DMARD (bDMARD) - single response

☐ Anti-TNF alpha   |   ☐ Anti-IL-6   |   ☐ Anti-CD20

Type of anti-TNF alpha - single response

   ☐ Infliximab   |   ☐ Etanercept   |   ☐ Adalimumab   |   ☐ Certolizumab   |   ☐ Golimumab

Spacing of biologic treatment - single response

☐ Yes   |   ☐ No

Corticosteroid therapy - single response

☐ Yes   |   ☐ No

Corticosteroid dose (mg prednisone equivalent): ______________________________________________

Treatment Tolerance and Adherence

Have you experienced intolerance to your disease-modifying treatment? - single response

☐ Yes   |   ☐ No

If yes, which treatment? - multiple responses possible

☐ Methotrexate   |   ☐ Sulfasalazine   |   ☐ Arava (leflunomide)   |   ☐ Anti-TNF alpha   |   ☐ Anti-IL-6   |   ☐ Anti-CD20

Treatment adherence was assessed using the four-item Morisky-Green-Levine Medication Adherence Questionnaire (MGL MAQ).

Score/result: ______________________________________________

Knowledge About Ramadan and Inflammatory Rheumatic Disease

Have you fasted during Ramadan since being diagnosed with rheumatoid arthritis? - single response

☐ Yes   |   ☐ No

If no, why? - multiple responses possible

☐ Inability to fast   |   ☐ Comorbidities   |   ☐ Beliefs/misconceptions   |   ☐ Other: __________

Have you ever asked your treating physician for information about fasting and inflammatory rheumatic disease? - single response

☐ Yes   |   ☐ No

Have you ever received information about your rheumatic disease and Ramadan fasting? - single response

☐ Yes   |   ☐ No

If YES, what was the source? - multiple responses possible

☐ Treating physician   |   ☐ Media   |   ☐ Word of mouth   |   ☐ Other: __________

Have you ever missed or declined a medical follow-up visit with your treating rheumatologist during Ramadan? - single response

☐ Yes   |   ☐ No

Use of antirheumatic treatment during Ramadan - single response

☐ I keep the same prescribed treatment   |   ☐ I reduce the dose myself without consulting my treating physician   |   ☐ I change the timing of treatment as recommended by my physician   |   ☐ I stop my treatment

At what time would you take your treatment during Ramadan?

Injectable methotrexate

☐ Iftar   |   ☐ Dinner   |   ☐ Suhoor   |   ☐ During the daytime fasting period   |   ☐ I stop my treatment

Oral methotrexate

☐ Iftar   |   ☐ Dinner   |   ☐ Suhoor   |   ☐ During the daytime fasting period   |   ☐ I stop my treatment

Leflunomide

☐ Iftar   |   ☐ Dinner   |   ☐ Suhoor   |   ☐ During the daytime fasting period   |   ☐ I stop my treatment

Sulfasalazine

☐ Iftar   |   ☐ Dinner   |   ☐ Suhoor   |   ☐ During the daytime fasting period   |   ☐ I stop my treatment

Corticosteroids

☐ Iftar   |   ☐ Dinner   |   ☐ Suhoor   |   ☐ During the daytime fasting period   |   ☐ I stop my treatment

Biologic treatment

☐ Iftar   |   ☐ Dinner   |   ☐ Suhoor   |   ☐ During the daytime fasting period   |   ☐ I stop my treatment

If you receive a methotrexate injection during the daytime fasting period, do you make up that fasting day? - single response

☐ Yes, because I consider it incompatible with fasting   |   ☐ No   |   ☐ Sometimes, when digestive intolerance is significant

If you receive an infusion of your biologic treatment during the daytime fasting period, do you make up that fasting day? - single response

☐ Yes   |   ☐ No

Based on your experience, do you consider fasting beneficial for your rheumatic disease?

Pain

☐ Yes   |   ☐ No   |   ☐ No difference

Morning stiffness

☐ Yes   |   ☐ No   |   ☐ No difference

Nocturnal awakenings

☐ Yes   |   ☐ No   |   ☐ No difference

Joint swelling

☐ Yes   |   ☐ No   |   ☐ No difference

Fatigue

☐ Yes   |   ☐ No   |   ☐ No difference

Beliefs and Perceptions

Can Ramadan fasting trigger a flare of my rheumatic disease? - single response

☐ Yes   |   ☐ No

Ramadan fasting is incompatible with having rheumatoid arthritis - single response

☐ Yes   |   ☐ No

Ramadan fasting is incompatible with taking antirheumatic treatment - single response

☐ Yes   |   ☐ No

I do not fast because of my comorbidities - single response

☐ Yes   |   ☐ No

Fasting improves my rheumatic disease - single response

☐ Yes   |   ☐ No

Pre-Ramadan Disease Activity and Quality-of-Life Assessment

Weight (kg): ______________________________________________

Height (m): ______________________________________________

BMI (kg/m²): ______________________________________________

Pain VAS: On a scale from 0 to 10, what was your level of pain this week?

☐ 0   |   ☐ 1   |   ☐ 2   |   ☐ 3   |   ☐ 4   |   ☐ 5   |   ☐ 6   |   ☐ 7   |   ☐ 8   |   ☐ 9   |   ☐ 10

Nocturnal awakenings: On a scale from 0 to 10, what was your level of nocturnal awakening this week?

☐ 0   |   ☐ 1   |   ☐ 2   |   ☐ 3   |   ☐ 4   |   ☐ 5   |   ☐ 6   |   ☐ 7   |   ☐ 8   |   ☐ 9   |   ☐ 10

Fatigue VAS: On a scale from 0 to 10, what was your level of fatigue this week?

☐ 0   |   ☐ 1   |   ☐ 2   |   ☐ 3   |   ☐ 4   |   ☐ 5   |   ☐ 6   |   ☐ 7   |   ☐ 8   |   ☐ 9   |   ☐ 10

Morning stiffness before Ramadan (minutes): ______________________________________________

Tender joint count before Ramadan: ______________________________________________

Swollen joint count before Ramadan: ______________________________________________

Erythrocyte sedimentation rate, first hour, before Ramadan: ______________________________________________

C-reactive protein (mg/L): ______________________________________________

DAS28 (to be calculated by the investigator): ______________________________________________

Post-Ramadan Assessment

Number of days fasted during Ramadan: ______________________________________________

Did you consult a physician from a specialty other than rheumatology during Ramadan? - single response

☐ Yes   |   ☐ No

Did you experience a non-rheumatologic problem during Ramadan that required consultation with another physician? - single response

☐ Yes   |   ☐ No

If yes, which specialty? - multiple responses possible

☐ General medicine   |   ☐ Gastroenterology   |   ☐ Pulmonology   |   ☐ Cardiology   |   ☐ Nephrology   |   ☐ Urology   |   ☐ Other: __________

During Ramadan, did you use treatments other than those prescribed by your treating physician? - single response

☐ Yes   |   ☐ No

If yes, which treatment?: ______________________________________________

Weight (kg): ______________________________________________

Height (m): ______________________________________________

BMI: ______________________________________________

Pain VAS: On a scale from 0 to 10, what was your level of pain this week?

☐ 0   |   ☐ 1   |   ☐ 2   |   ☐ 3   |   ☐ 4   |   ☐ 5   |   ☐ 6   |   ☐ 7   |   ☐ 8   |   ☐ 9   |   ☐ 10

Nocturnal awakenings: On a scale from 0 to 10, what was your level of nocturnal awakening this week?

☐ 0   |   ☐ 1   |   ☐ 2   |   ☐ 3   |   ☐ 4   |   ☐ 5   |   ☐ 6   |   ☐ 7   |   ☐ 8   |   ☐ 9   |   ☐ 10

Fatigue VAS: On a scale from 0 to 10, what was your level of fatigue this week?

☐ 0   |   ☐ 1   |   ☐ 2   |   ☐ 3   |   ☐ 4   |   ☐ 5   |   ☐ 6   |   ☐ 7   |   ☐ 8   |   ☐ 9   |   ☐ 10

Morning stiffness after Ramadan (minutes): ______________________________________________

Tender joint count after Ramadan: ______________________________________________

Swollen joint count after Ramadan: ______________________________________________

Erythrocyte sedimentation rate, first hour, after Ramadan: ______________________________________________

C-reactive protein (mg/L) after Ramadan: ______________________________________________

DAS28 after Ramadan (to be calculated by the investigator): ______________________________________________

During Ramadan, did you increase the dose of your symptomatic treatment because you felt worse? - single response

☐ Yes   |   ☐ No

If yes, which treatment?: ______________________________________________

Dose: ______________________________________________

Treatment adherence was assessed using the four-item Morisky-Green-Levine Medication Adherence Questionnaire (MGL MAQ).

Score/result: ______________________________________________
